# Cardiovascular disease and high blood pressure trend analyses from 2002 to 2016: after the implementation of a salt reduction strategy

**DOI:** 10.1186/s12889-018-5634-z

**Published:** 2018-06-11

**Authors:** D. Abreu, P. Sousa, C. Matias-Dias, F. J. Pinto

**Affiliations:** 1 Escola Nacional de Saúde Pública, Universidade Nova de Lisboa, Avenida Padre Cruz, 1600-560 Lisboa, Portugal; 2 Centro de Investigação em Saúde Pública - ENSP-UNL, Avenida Padre Cruz, 1600-560 Lisboa, Portugal; 3 Department of Epidemiology of the Instituto Nacional de Saúde Doutor Ricardo Jorge, Avenida Padre Cruz, 1649-016 Lisboa, Portugal; 4 Serviço de Cardiologia, Hospital de Santa Maria, Centro Hospitalar Lisboa Norte - EPE, Centro, Académico Medicina de Lisboa, Lisbon, Portugal; 5 Centro Cardiovascular da Universidade de Lisboa, Av. Prof. Egas Moniz, 1649-035 Lisboa, Portugal

**Keywords:** Cardiovascular disease, High blood pressure, Population wide-approach, Public health

## Abstract

**Background:**

Cardiovascular disease (CVD) is the leading cause of death around the world; however, many CVD events could be prevented if we focused on modification of the main risk factors.

Increased salt consumption is estimated to have caused millions of deaths, mostly related to CVD, particularly stroke, which is the leading cause of death in Portugal.

In our study, we aim to assess trends in the proportion of high blood pressure (HBP) in Acute Coronary Syndrome (ACS) patients as well as the trends in stroke and ACS in Portugal, especially after a set of public health initiatives were implemented to reduce salt intake.

**Methods:**

The monthly proportion of ACS patients presenting with previously diagnosed HBP and the monthly rate of CVD admissions into public hospitals in Portugal were calculated. CVD rates were stratified into ACS rate and stroke rates. Data were stratified by demographics variables. An interrupted time-series model was used to assess changes over time.

**Results:**

Breakpoint analysis revealed an estimated breakpoint around the year 2013 for the proportion of HBP patients, the following year there was a decreasing trend, however it was not significant. Analyses showed the trend before 2013 was increasing and started to decrease after this year. This decreased in proportion of HBP patients can be translated into a reduction of 555 people per year presenting with HBP in the ACS population.

We analysed trends for ACS and stroke and tested the significance for a breakpoint in the year 2013. Although none of the remaining trends were significant for ACS crude rates and stroke crude rate, a decreasing trend was observed.

**Conclusions:**

This research provides an indication about the impact a population-wide approach to CVD risk factors has on CVD trends themselves. Our results suggest that population-wide approaches can have an impact on the prevention and improvement of CVD control, reducing the number of CVD events, and eventually reducing premature death by CVD. As more restrictions on salt intake are being planned in Portugal in the next years, it is highly relevant to assess what is the current panorama and what further reductions we can expect.

## Background

Cardiovascular disease (CVD) is the leading cause of death around the world [1]. An estimated 4 million people in Europe die by CVD annually [2]. However, many of the CVD events could be prevented if we focused on modification of the main risk factors [3].

In 2010, high blood pressure (HBP) was the leading risk factor contributing to global disease burden, accounting for more than 15% of all health loss in adults [4], and responsible for 62% of all strokes and 49% of ACS events [5]. Although sodium is an essential nutrient necessary for maintenance of plasma volume, acid-base balance, transmission of nerve impulses, and normal cell function [6], current salt consumption is much greater than needed for survival, creating an overload on the metabolic system [7]. This overload can increase blood pressure (BP) [8]. Increased salt consumption is estimated to have caused millions of deaths, mostly related to CVD, particularly stroke, which is the leading cause of death in Portugal [9], being one of the countries with the highest mortality rates by stroke among the Western countries [10]. The high prevalence of HBP is pointed to as one of the main reasons.

Implementing both population-wide and high-risk approaches to reduce blood pressure seems almost inevitable due to the large burden of HBP [11, 12]. Even small reductions of HBP prevalence in the population could lead to great health gains. Knowing the importance of these approaches, the World Health Organization (WHO) created a set of recommendations to reduce dietary salt to 5 g/day, in order to prevent chronic disease and improve health [13]. In the European Union, 26 out of the 53 Member States, including Portugal, implemented operational salt reduction policies including laws aiming to reduce salt intake. Recent reports have shown that salt consumption in Portugal has been declining in recent decades to a minimum of 7.3 g/day in 2016 [14]. Prospective studies have shown that reducing salt intake can lead to reductions in blood pressure and, eventually, to a reduction in CVD events [15].

By 2010 a set of public health initiatives were implemented in Portugal, namely a reduction in salt added to bread and mandatory salt labelling in pre-packed food. As these initiatives are meant to reduce salt intake we hypothesized that it could lead to a reduction in the proportion of HBP and CVD, namely ACS and stroke. Therefore in our study, we aim to assess the trends in the proportion of HBP in ACS patients, as well as the trends in stroke and ACS in the country, before and after these initiatives were applied. For the sake of clarity in our study we refer as salt for all sodium data.

## Methods

### Study population

Two population sets were used in this study. Approval to access data was obtained previously. For both sets of data, participants included in the study were over 20 years old.

### First population dataset

The National Registry of Acute Coronary Syndrome (NRACS) [16]. All data from 2002 to 2015 were extracted for our study. This dataset was used to obtain the proportion of ACS patients previously diagnosed with HBP. This register collects information for ACS only, not just for risk factors but also demographics.

This dataset has the advantage of being integrated into the Euro Heart Survey platform and, consequently, uses the Cardiology Audit and Registration Data Standards (CARDS) system. This system ensures that credible and comparable information is collected in several European countries over time as they use standardised information, both in terms of the definition and coding of variables, and in the form of measurement and collection of data. As this data is validated and standardised, it can be applied to and used to validate analyses in other larger populations, thus being able to obtain more robust results [17]. HBP in this dataset was defined as previously diagnosed by a physician, or known blood pressure > 140 mmHg systolic or > 90 mmHg diastolic on two or more occasions.

### Second population dataset

The National database that collects data from all admissions into Portuguese public hospitals (Mainland Portugal). Data from 2002 to 2016 were extracted for our study. This database uses the Diagnosis Related Group (DRG) system holding data on primary diagnosis and some demographic variables, such as sex and age, as well as the geographic region of admission [18]. DRG codes for ischemic and haemorrhagic stroke were included in the analysis (ICD 9th codes for stroke: 43301, 43321, 43311, 43331, 43391, 43401, 43411, 43491, 431, 432, 4320, 4321, 4329, and 43381) as well as codes for ACS (ICD 9th codes: 410.00–410.xx to identify admissions diagnosed as Acute Myocardial Infarction and code 4130 to identify unstable angina).

The two datasets were analysed separately as there is no possible method to identify the participants in the dataset, thus the same participant can in fact be registered in both datasets, however as the outcomes studied are independent from each other we do not expect this fact to influence on the validity of the analysis. Similarly the fact that the two datasets used in our study are carefully monitored by the Portuguese Society of Cardiology, in the case of the NRACS, and the Directorate of General Health, in the case of the DRG data, ensures the validity of these data.

### Statistical analysis

Two main outcomes were analysed in our study, the monthly proportion of ACS patients presenting with previously diagnosed HBP and the monthly rate of CVD admission into public hospitals in the country. CVD rates were stratified into ACS rate and stroke rates.

The proportion of ACS patients with HBP was obtained by dividing the number of monthly HBP diagnoses by the total ACS patients registered for month.

We applied an interrupted time series design, implementing a segmented multiple linear regression model, in which the response variable was the monthly the proportion of ACS patients with HBP. These models are useful when the relationship between the response and the independent variables are piecewise linear, namely represented by two or more straight lines connected at unknown values, which are usually referred to as breakpoints. In our case, the breakpoint would be expected for any given year where there was a change in the trend of the proportion of patients with HBP.

R “segmented” package was used to determine the presence of any breakpoints in the trends found.

Crude event rates for ACS and stroke (per 100,000 adults) were computed for each month, using the population of the country, restricted to the population resident in mainland Portugal, with ages over 20 years as the denominator. Crude rates were calculated as the number of events, for each outcome separately, divided by the Portuguese population for that month.

As the effect of this type of population wide approaches might not be immediate [19, 20], taking some time to show effects in the population, we used the estimated breakpoint from the monthly proportion of ACS patients with HBP as a proxy for the effect of the estimated breakpoint in CVD trends.

The impact of the breakpoint estimated on the CVD trends, was assessed through a multiple linear regression model using standard methods for interrupted time series.

We included one dichotomous variable that accounted for the main effect on hospital admissions of the estimated breakpoint for the proportion of HBP patients and an interaction between the breakpoint estimated and time, to evaluate changes over time following the breakpoint.

This model was implemented in order to test whether there was a significant change in the number of events, and if there was any change in the proportion of HBP patients was observed.

A month indicator covariable was introduced to adjust for seasonality in the outcomes admissions.

All analyses were stratified by sex and age. Age was grouped into two categories < 65 and ≥ 65 years old. Autocorrelation between month estimates was incorporated adequately into the model, with the presence of short term autocorrelation applying a first order autoregressive - AR (1) - structure to the residuals. Models were fitted in R version 2.3.2 software.

Statistical significance was assessed through *p*-values, assuming < 5% as significant and 95% confidence intervals (CI) were calculated for each of the regression coefficients.

## Results

**Fig. 1:**
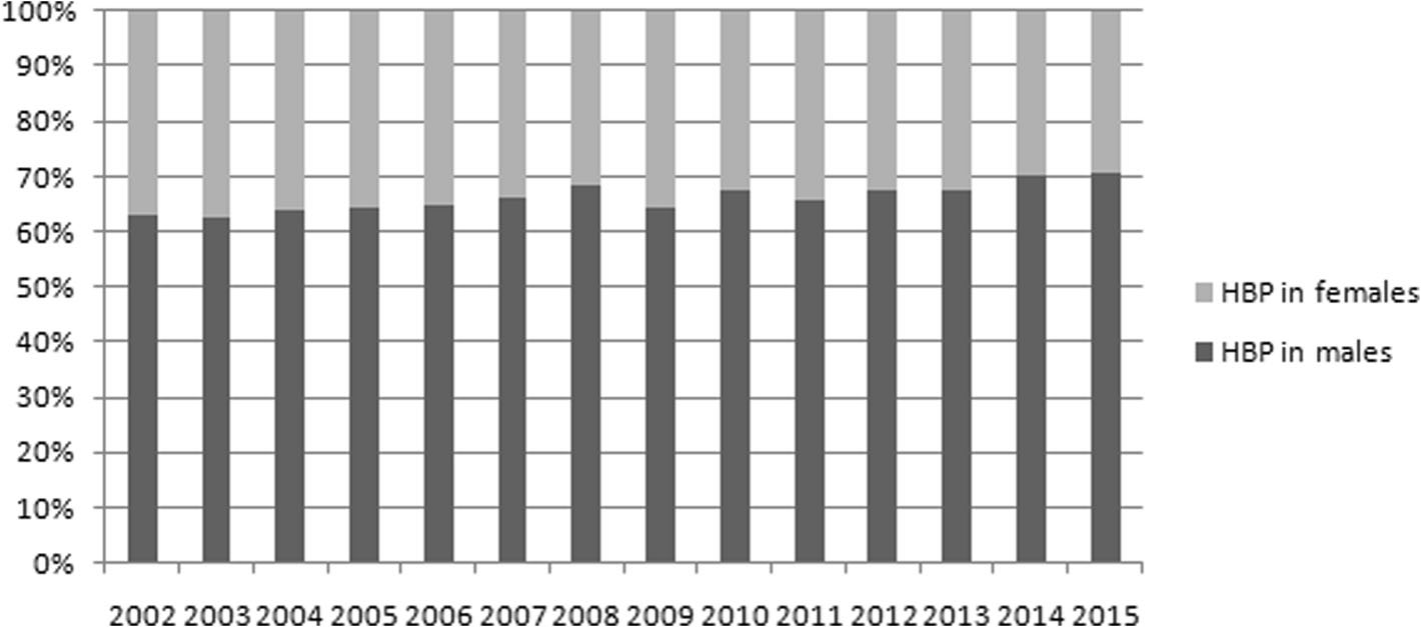
Proportion of patients with HBP in the ACS population. Female/male ratio from years 2002 to 2015

A total of 43,271 ACS events were registered in the NRACS over the last 14 years. The proportion of males presenting with ACS has been consistently higher than women, with around 69% of ACS events occurring in males. Mean age through the years of study has also been fairly steady, ranging from 65 to 66 years old (Table 1). Male/female ratio for HBP patients (Fig. 1) has also remained fairly steady through the years in the ACS population.

**Table 1:** Demographic characterization of the data analysed

	ACS (DRG data)	Stroke (DRG data)	HBP in ACS patients (NRACS data)
Before regulation			
*Female %*	26,00%	49,43%	35,33%
*Age ≥ 65%*	37,93%	77,15%	65,38%
After regulation			
*Female %*	26,36%	49,61%	32,67%
*Age ≥ 65%*	37,85%	78,26%	64,29%

The proportions of patients with HBP ranged from a minimum proportion of 50.0% to a maximum of 79.2%.

**Fig. 2:**
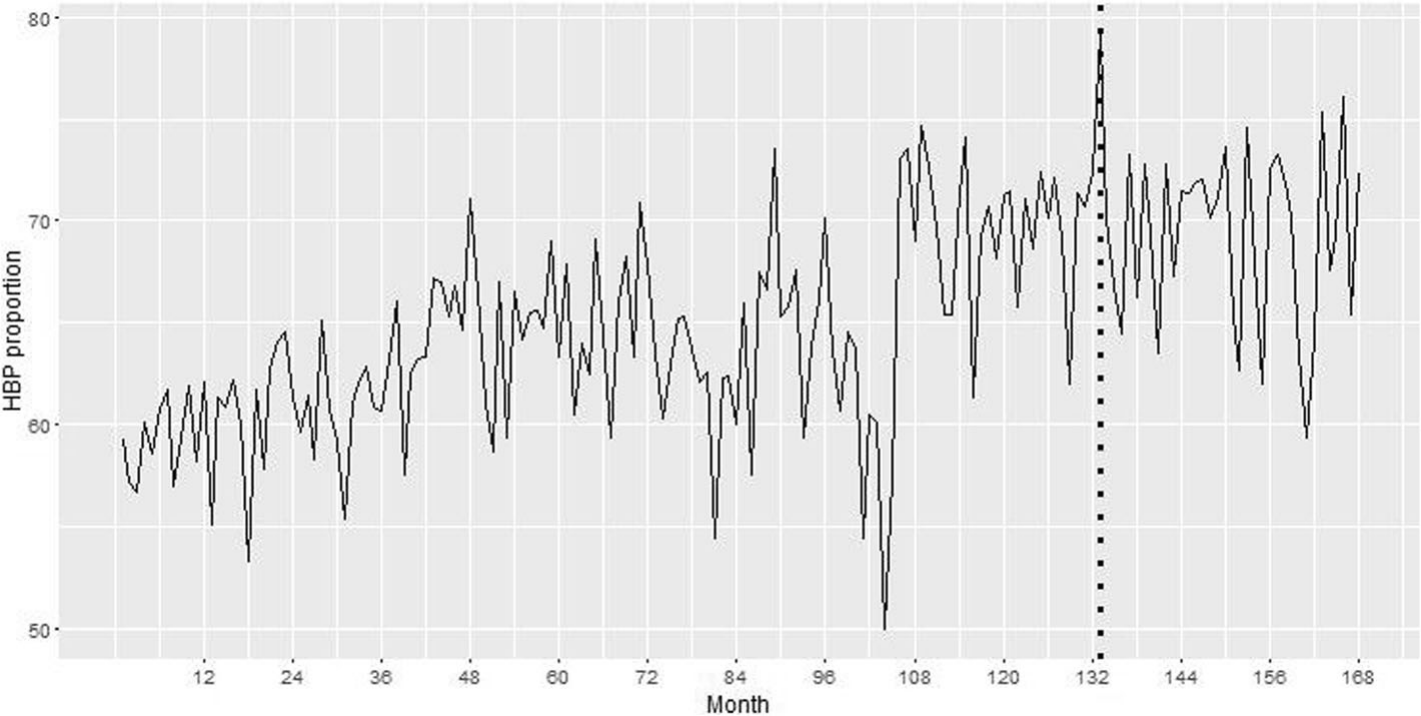
Trends in overall monthly proportion of HBP in ACS patients from January 2002 to December 2015. Dashed line represents the beginning of 2013, year associated to the estimated decline in trends

Breakpoint analysis revealed an estimated breakpoint around the year 2013 for the proportion of HBP patients (Fig. 2), the year after there is a decreasing trend, however it was not significant (*p*-value = 0.832). Analyses showed the trend before 2013 was, in fact, increasing and started to decrease after this year (β_before_ = 0.009, CI: 0.007, 0.011; β_after_ = − 0.003, CI: -0.037, 0.0302). Although zero is present in the CI for the slope after 2013, the change between both slopes (before and after 2013) is negative with a decrease of − 0.012 in the monthly proportion. Thus, the decrease estimated after 2013 is higher than the increase estimated before 2013. This decrease in proportion can be translated into a reduction of 555 people per year presenting with HBP in the ACS population (Table 2).

**Table 2:** Results from the three regressions applied, segmented multiple linear regression model, for the proportion of HBP patients, and both multiples linear regressions using standard methods for interrupted time series, for the ACS and stroke outcomes

	β (CI)	*t*-value	*p*-value
HBP proportion			
Overall^a^			
Pre-breakpoint trend (change per month)	0.004 (0.003;0.006)	–	–
Change in trend (post-breakpoint vs pre-breakpoint)	−0.006	–	< 0.001
Post-breakpoint trend (change per month)	−0.002 (− 0.003;-0.001)	–	–
Male^a^			
Pre-breakpoint trend (change per month)	− 0.003(− 0.022;0.022)	–	–
Change in trend (post-breakpoint vs pre-breakpoint)	− 0.016	–	0.215
Post-breakpoint trend (change per month)	−0.019(− 0.061;0.023)	–	–
Female^a^			
Pre-breakpoint trend (change per month)	−0.003(− 0.028;0.022)	–	–
Change in trend (post-breakpoint vs pre-breakpoint)	− 0.016	–	0.215
Post-breakpoint trend (change per month)	−0.018(− 0.052;0.014)	–	–
Age < 65^a^			
Pre-breakpoint trend (change per month)	−0.022(− 0.798;0.754)	–	–
Change in trend (post-breakpoint vs pre-breakpoint)	0.011	–	> 0.05
Post-breakpoint trend (change per month)	−0.011(− 0.039;0.017)	–	–
Age ≥ 65^a^			
Pre-breakpoint trend (change per month)	0.029(−0.017;0.075)	–	–
Change in trend (post-breakpoint vs pre-breakpoint)	−0.018	–	> 0.05
Post-breakpoint trend (change per month)	0.011(−0.017;0.039)	–	–
ACS crude rates (per 100,000 adults)			
Overall^a^			
Time of the breakpoint	−0.112(− 2.041;1.817)	−0.114	0.909
Time of the breakpoint^a^ time interaction	−0.031(− 0.09;0.036)	−0.903	0.368
Male^a^			
Time of the breakpoint	0.289(− 2.269;2.848)	0.222	0.825
Time of the breakpoint^a^ time interaction	−0.018(− 0.108;0.072)	−0.393	0.695
Female^a^			
Time of the breakpoint	−0.497(− 1.927;0.932)	−0.682	0.496
Time of the breakpoint^a^ time interaction	0.013(−0.034;0.060)	0.560	0.576
Age < 65^a^			
Time of the breakpoint	0.151(−0.723;1.024)	0.338	0.736
Time of the breakpoint^a^ time interaction	−0.015(− 0.044;0.015)	−0.986	0.325
Age ≥ 65^a^			
Time of the breakpoint	0.151(− 5.159;6.721)	0.258	0.7970
Time of the breakpoint^a^ time interaction	−0.015(− 0.358;0.066)	−1.349	0.1792
Stroke crude rates (per 100,000 adults)			
Overall^a^			
Time of the breakpoint	0.265(−1.399;1.929)	0.312	0.755
Time of the breakpoint^a^ time interaction	−0.037(− 0.091;0.016)	−1.370	0.172
Male^a^			
Time of the breakpoint	0.900(−0.743;2.543)	1.074	0.284
Time of the breakpoint^a^ time interaction	−0.022(− 0.074;0.030)	−0.822	0.412
Female^a^			
Time of the breakpoint	0.999(−1.204;3.202)	0.888	0.376
Time of the breakpoint^a^ time interaction	−0.047(− 0.121;0.027)	−1.244	0.215
Age < 65^a^			
Time of the breakpoint	0.448(−0.261;1.157)	1.238	0.217
Time of the breakpoint^a^ time interaction	−0.014(− 0.037;0.009)	−1.207	0.229
Age ≥ 65^a^			
Time of the breakpoint	2.548(− 5.351;10.446)	0.632	0.528
Time of the breakpoint^a^ time interaction	−0.238(− 0.502;0.026)	−1.764	0.079

Values not presented in the table (−) are not available for the type of regression

β represents the coefficients in the regression

*HBP* High blood pressure, *ACS* Acute coronary syndrome, *CI* confidence interval

^a^All models were adjusted for seasonality

**Fig. 3:**
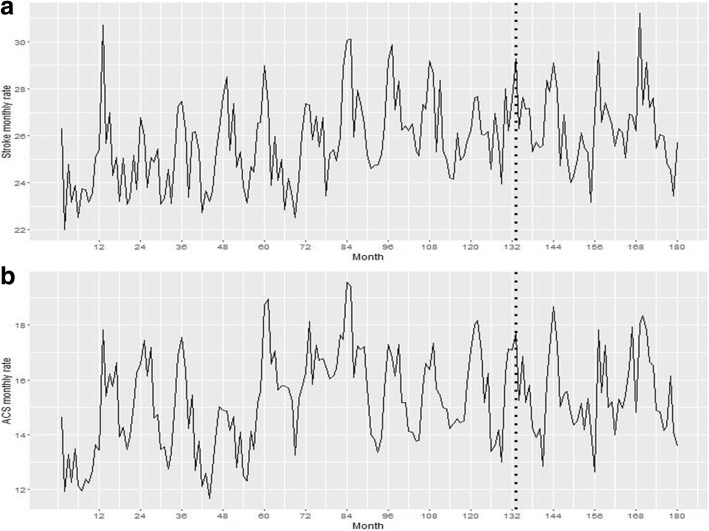
Trends in overall monthly crude rates of CV admissions from January 2002 to December 2016 per 100,000 adults. a) Trends for stroke crude rates admissions. b) Trends for ACS crude rates admissions. Dashed lines represent the beginning of 2013, year associated to the estimated decline in trends

Figure 3 displays the frequency of ACS and stroke admissions from 2002 to 2016. A total of 115 public hospitals from mainland Portugal registered ACS admissions from 2002 to 2016 and 122 registered stroke admissions. A maximum age of 99 years old was found in the database and the majority of admissions were for both ACS and stroke.

We analysed trends for ACS and stroke and tested the significance for a breakpoint in the year 2013.

Although none of the remaining trends were significant (Table 2) for ACS crude rates (β = − 0.057, CI: - 0.154, 0.039) and stroke crude rate (β = − 0.049, CI: -0.128, 0.029), a decreasing trend can be observed.

When further stratified data by sex and age, although no significant trends were observed, a decreasing trend for ACS was found in women but not in men. The largest decrease was found for people over 65 years old for both outcomes. Nevertheless, all these results should be interpreted with caution as none of the coefficients were significant (*p*-values > 5%), and zero was always included in the confidence interval.

The seasonal pattern observed for ACS and stroke was consistent with that reported elsewhere [21], with higher rates of admission over winter, and lower rates during the summer (Fig. 3).

## Discussion

Our results showed increasing trends in the proportion of HBP comorbidity in the ACS population until 2013, the following years the trends appear to decrease. This year was used as a breakpoint to test for differences in trends for ACS and stroke. Decreasing trends for both outcomes were also found, but were not significant.

Although our results were not statistically significant, and thus must be interpreted with caution, it is encouraging to find decreasing trends, especially in the proportion of HBP patients in the ACS population, with a reduction of nearly 600 people per year, after the implementation of a major public health measure that was meant to reduce salt intake.

Even a small reduction in the proportion of HBP in the ACS population, as the one observed in our study of 555 persons per year achieved through a population-wide approach, can have huge impacts [22, 23]. This impacts has effectively slowed down the development of atherosclerosis in young people, thereby reducing the likelihood of future epidemics of CVD [24].

The decreasing trend for ACS was observed for women but not in men, in fact salt intake have been previously link to higher risk of CV events in women but not in men [25], more particularly studies have found that women could benefit more, concerning stroke reduction, from dietary salt reduction than men [26].

As salt sensitivity is known to be greater in the elderly we hypothesized that if the salt reduction law could have some effect it would be greater for the elderly [27, 28]. In fact our results suggest a decreasing trend for all outcomes in the older group.

Results from this type of population-wide approach were seen in the UK, where salt reduction campaigns showed significant results, achieving a 15% salt reduction from 2003 to 2011, which translated into about 6000 fewer deaths from CVD, saving about 1.5 billion pounds a year [8].

The decreasing trends for all of our outcomes was not as great as we expected, however the fact that the starting of the decrease takes place after the implementation of these approaches, and the trend of HBP in ACS patients was steadily increasing before that, supports our hypothesis that this approach of reducing salt in bread and making pre-packed salt labelling mandatory can influence HBP prevalence in ACS patients as well as CVD trends.

The fact that, salt intake levels have been decreasing in Portugal to the most recent value of 7.3 g/day in 2016 [14] also supports our hypothesis of the potentially effect of the regulation applied in the country. Although this value was obtained by a 24 h dietary recall questionnaire [29], these values were validated against urinary sodium excretion for a sub-sample of 100 subjects and the values were highly correlated.

In addition, BP levels have been declining in the Portuguese population, from a mean BP of 134.7/80.5 mmHg in 2003 [30] to a mean BP of 127/74.6 mmHg in 2012 [10].

Furthermore, several studies showed that bread is one important source of salt in Portugal [7], and contributes to about one-sixth of daily salt intake [7]. Results from the National Food, Nutrition and Physical Activity Survey suggest most of the salt consumed by the population comes directly from bread and toast, charcuterie products, and soups [14]. It was all this evidence that led to the creation of the regulation to reduce salt in bread to a minimum of 1.4 g of salt per 100 g of final product [31] and mandatory labelling for pre-packed products stating clearly the salt content of the product. Portugal was, thus, the first western country to create a law for the clear definition of the quantity of salt contained in bread.

Besides the legislation in 2010, several initiatives have been carried in Portugal to reduce salt intake, such as: electronic tools, such as websites included in the National Program for the Promotion of Healthy Eating, to free distribution of books and brochures, by the creation of an animated series, and the creation of the National Food, Nutrition and Physical Activity Survey, aiming to collect nationwide data on dietary intake and physical activity [14].

There is already evidence that reducing salt intake could reduce BP in the Portuguese population. One community intervention trial conducted in Portugal demonstrated that reducing salt intake in an entire village, including cooking and processed food, led to a significant reduction in the BP of the population [32].

Policy interventions to reduce national sodium consumption have demonstrated to be highly cost effective in nearly every country in the world. These interventions could reduce millions of disability adjusted life years at low cost and be more cost-effective than pharmacological interventions [26, 33].

Policy and system changes are critical to reduce HBP in populations, including legislation and public education to reduce dietary sodium and food pricing policies, to support prevention and management of CVD [34].

Alternative public health approaches, such as reducing salt in processed foods and bread, and labelling of processed food along with the use of multiple fiscal and educational policies, have already been proposed by the WHO as the first-line approach for CVD reduction, when implemented on a wide scale [24, 34].

In spite of several efforts being made in the country to reduce salt intake, there is still room for improvement, namely in food labelling [35], adjusting it to the level of health literacy in the country. Once the level of health literacy in Portugal was slightly lower than the rest of European countries [36], this might have prevented the population from fully benefiting from the labelling, as the interpretation of salt content in each food can be difficult.

Simpler labelling systems already exist in other countries, such as labels that rely on the use of front-of-pack information such as logos with information on the overall healthiness of the food and traffic light and colour coding systems, which might help citizens make healthier choices. Countries such as Finland and the UK have already implemented some of these labelling systems. In Finland, food products with salt contents below the designated levels display a low-salt label to emphasise their lower-than-conventional salt levels [37]. In the UK, a traffic light system to distinguish between high, medium, and low salt content in food products was implemented. These strategies proved to be efficient in reducing salt intake in the population [38]. The traffic light labelling system has been proposed by the Portuguese Hypertension Society to help the population choose products regardless of their level of literacy.

More restrictions are being planned in Portugal to further reduce salt intake, such as a taxes on products with more than 1 g of salt per 100 g of finished product such as: pre-packaged crackers and biscuits; pre-packaged cereal flakes and pressed cereals; and pre-packaged, dehydrated potato chips. A rate of 0.80 Euros per kilogram of finished product will be applied to these products.

On the other hand, further reduction of salt content in bread, to a minimum of 1 g per 100 g of final product, was proposed to be achieved by 2020, with continuous reductions each year after 2018.

Our study presents encouraging indications suggesting there have been reductions in the proportion of hypertensives among ACS patients after 2013, along with an annual decrease in the rates of ACS and stroke.

### Limitations

However, we recognise some limitations of the study. Like any ecological study, it is not possible to prove causal relations, i.e. direct association between the initiatives to reduce salt implement in the country the reduction observed in the proportion of hypertensives ACS patients. However, our methodology was applied, using the same data to analyse the effects of the smoking ban legislation on ACS trends, and a significant reduction was found right after the ban [39].

On the other hand, it might be too soon to notice great effects from salt reduction on CVD trends.

The fact that until 2013, the trend for the proportion of hypertensives was continuously increasing, and right after 2013 a decrease in trend can be observed, suggests that the lack of statistical significance could result from the time period being analysed being too short to capture further reductions.

Moreover, our study has some strengths, such as the fact that we used two well validated and standardised databases is a major strength as this allows for an easy comparison with other international studies mainly those developed in other European countries. As well as the availability of information on gender and age, this allowed us to assess the robustness of our findings among different subgroups. Also the time series method is preferred over the simpler pre- and post-proportion comparison method that does not take the pre-intervention trend into account, and also allows correction for autocorrelation [40].

## Conclusions

In conclusion, the fact that we found decreasing trends for both HBP in ACS patients and CVD outcomes after major initiatives to reduce salt intake were implemented in the country, suggests that this type of approaches, although hard to measure, can impact the trends of major public health outcomes. Thus, this research is relevant to the public health community because it provides an indication about the impact that a population-wide approaches can have on CVD risk factors, and CVD trends themselves. Our results suggest that population-wide approaches can have an impact on the prevention and improvement of CVD control, reducing the number of CVD events and eventually reducing premature death, morbidity, and disability by CVD. Our study also highlights the importance of measuring the impact of these types of initiatives. These findings stress the need for public health policy in CVD to improve the health of populations addressing, in the first place, the well identified risk factors for CVD.

Undoubtedly, more studies are needed to assess the impact of such measures, not only for other diseases beside CVD, but also adding more years to the study would allow analysis of long terms effects of the public health initiatives. On the other hand, as more restrictions on salt intake are being planned in Portugal, in the next years it is very relevant to assess what is the current panorama and what further reductions we can expect.

## Abbreviations

ACS: Acute Coronary Syndrome
BP: Blood pressure
CI: Confidence interval
CVD: Cardiovascular disease
DRG: Diagnosis related group
HBP: High blood pressure
NRACS: National Registry for Acute Coronary Syndrome
